# Perceived shift of the centres of contracting and expanding optic flow fields: Different biases in the lower-right and upper-right visual quadrants

**DOI:** 10.1371/journal.pone.0211912

**Published:** 2019-03-07

**Authors:** Xiaorong Cheng, Chunmiao Lou, Xianfeng Ding, Wei Liu, Xueling Zhang, Zhao Fan, John Harris

**Affiliations:** 1 School of Psychology, Central China Normal University, Wuhan, China; 2 Key Laboratory of Adolescent Cyberpsychology and Behavior (CCNU), Ministry of Education, Wuhan, China; 3 Key Laboratory of Human Development and Mental Health of Hubei Province, Wuhan, China; 4 School of Psychology and Clinical Language Sciences, The University of Reading, Whiteknights, Reading, United Kingdom; University of Alberta, CANADA

## Abstract

We studied differences in localizing the centres of flow in radially expanding and contracting patterns in different regions of the visual field. Our results suggest that the perceived centre of a peripherally viewed expanding pattern is shifted towards the fovea relative to that of a contracting pattern, but only in the lower right and upper right visual quadrants and when a single speed gradient with appropriate overall speeds of the trajectories of the moving dots was used. The biases were not systematically related to differences of sensitivity to optic flow in different quadrants. Further experiments demonstrated that the biases were likely due to a combination of two effects: an advantage of global processing in favor of the lower visual hemifield and a hemispheric asymmetry in attentional allocation in favor of motion-induced spatial displacement in the right visual hemifield. The bias in the lower right visual quadrant was speed gradient-sensitive and could be reduced to a non-significant level with the usage of multiple speed gradients, possibly due to a special role of the lower visual hemifield in extracting global information from the multiple speed gradients. A holistic processing on multiple speed gradients, rather than a predominant processing on a single speed gradient, was likely adopted. In contrast, the perceived bias in the upper right visual quadrant was overall speed-sensitive and could be reduced to a non-significant level with the reduction of the overall speeds of the trajectories. The implications of these results for understanding motion-induced spatial illusions are discussed.

## Introduction

There has been continuing interest in the interaction between the mechanisms of motion perception and those of spatial localization. A consistent finding has been that the static edge of a window containing unidirectional motion is displaced perceptually in the direction of the motion [1–14], whether this is produced by physical movement or by the apparent motion of the movement aftereffect.

In natural images, edges are often defined by the relationship between differences in the direction of motion in two regions, not simply that between a static and a moving region. In previous work, we investigated the apparent displacement of a vertical contour defined by two abutting regions of translating dots moving in opposite directions [15, 16]. When fixated directly, such contours are localised accurately, but when presented in peripheral vision, they appear displaced, in a direction that depends on the relative directions of motion and on the quadrant of the visual field in which they were presented. In the lower right visual quadrant, a contour defined by a diverging pattern was perceived as shifted away from the fovea relative to a contour defined by a converging pattern. However, an opposite pattern of shifts was observed in the upper left visual quadrant, in that the contour defined by divergence was perceived as closer to the fovea than one defined by convergence.

We suggested that these anisotropies in the spatial localization of motion-defined contours depended on the relative strengths of two effects. One is the perceived expansion of an area in centripetal motion, stemming from a greater sensitivity to this direction of motion [17–19]. This centripetal bias is only observed in the lower, not the upper, visual field, which can explain why significant displacements were not present in the upper right quadrant. The other effect is the asymmetry in the allocation of spatial attention [20], in which the left hemifield is favoured. We suggested that attention can act to boost centrifugal signals, to compensate for the anisotropy between centripetal and centrifugal motion. In the lower left quadrant the two effects cancel, so producing no significant displacements, whereas in the upper left quadrant, in which there is little centripetal bias, the boosting of centrifugal signals leads to the opposite pattern of displacements to that in the lower right quadrant.

In all studies to date, spatial displacements have been mainly produced by unidirectional motions, such as translation toward a single direction, but see Whitney, et al. [11] and Liu, Ashida, Smith, & Wandell [21]. In explaining how perceived displacements might occur in our opponent motion stimuli, we suggested a local process comparing the outputs of overlapping motion-sensitive receptive fields that spanned the contour [15]. In the present study, we examine possible displacements of the centres of radially expanding and contracting patterns. We had two motivations for this. First, it is likely that translation and expansion/contraction are processed by different neural mechanisms, the former is processed as early as in V1, the latter starts from extrastriate regions such as MT [22–25]. It could be that the inputs to extrastriate mechanisms from V1, distorted by the biases we observed for simple translation, cause an apparent displacement in the centre of expansion/contraction, or that either or both biases may have no effect on MT. Thus it is not clear whether the same pattern of spatial displacement will exist for radially changing stimuli. One reason to expect a difference comes from a neuropsychological study by Vaina and colleagues [26]. In this single-case study, the female patient displayed a functional dissociation between local and global motion processing. For instance, her performance on a task requiring local motion processing, such as indicating the presence of a motion-defined contour, was extremely impaired. In contrast, on tasks involving global motion integration, such as measurement of motion coherence thresholds, she performed similarly to a normal control group. We argue in the General Discussion that the discrimination of centres of flow, unlike the localization of motion-defined contours, is reliant on more global information.

The second motivation for the present study arises because the radially expanding/contracting optic flow patterns used in the current study are ecologically meaningful in many situations, such as running, walking and driving. A recent EEG study [27] demonstrated that human infants’ brain electrical activities were sensitive to the radially expanding/contracting optic flow patterns that simulated forward and backward self-motion in depth relative to non-structured randomly moving dots as early as an age of 11–12 months. This is likely due to the fact that human infants become more and more mobile during the first year of life and develop adequate experience on the expanding/contracting optic flow patterns via self-produced locomotion [27]. Many studies [28–30] have supported the view that the human visual system can extract directional information from such patterns during movement of the observer. Any systematic perceived displacements of the centres of contracting or expanding optic flow would have possible implications for the accuracy of perceived heading.

In all experiments in the present study, the four visual quadrants were tested separately. In Experiment 1, the displays were what would be produced by smooth movement towards or away from a single fronto-parallel surface, and the relative positions of their centres were judged. Experiment 2 measured sensitivity to discriminate positions of the centres of expanding and contracting patterns, partly to establish whether this might underlie any perceived displacements. In Experiment 3, perceived displacements were measured for displays changed to those that would be produced by movement towards or away from multiple fronto-parallel surfaces. Experiment 4 & 5 were two control experiments to further explore the role of the overall speeds of the trajectories of the moving dots and the role of the average dot density in the pattern of perceived shift and the precision of discrimination of the centres of contracting and expanding flow patterns.

## Experiment 1

If the centres of radially changing patterns are perceived as displaced in the same way as contours defined by opposite directions of translation, we would expect the centre of the expanding pattern to appear further from the fovea than that of the contracting pattern in the lower right quadrant. Conversely, the centre of the contracting pattern would appear further away in the upper left quadrant. In Experiment 1, we tested this idea by presenting expanding and contracting patterns in all four visual quadrants separately, and exploring whether the perceived displacement of the centre of flow was significantly different from zero in any visual quadrant.

### Materials and methods

A total of 20 right-handed observers, 16 females and 4 males, aged from 19 to 24, took part in the experiment. All observers were university students, naïve to the aim of this research, and were paid for their participation. All observers had normal or corrected to normal vision, with no history of visual disorders. This and subsequently reported experiments had been approved by the local Research Ethics Committee, and observers gave their informed consent to participate.

#### Apparatus

The participant was seated in a room that was dark except for the display. The displays in this experiment were programmed in MATLAB (Mathworks Inc.) and Psychophysics Toolbox [31, 32], at a spatial resolution of 1024 x 768 pixels and were displayed on a 17" IIYAMA CRT monitor. Observers’ responses were recorded via a keyboard connected to the PC. The viewing distance between the centre of the screen and the mid-point of the observer’s eyes was 57cm. The position of the observer’s head was held constant by a chin rest.

#### Stimuli

**Fig 1:**
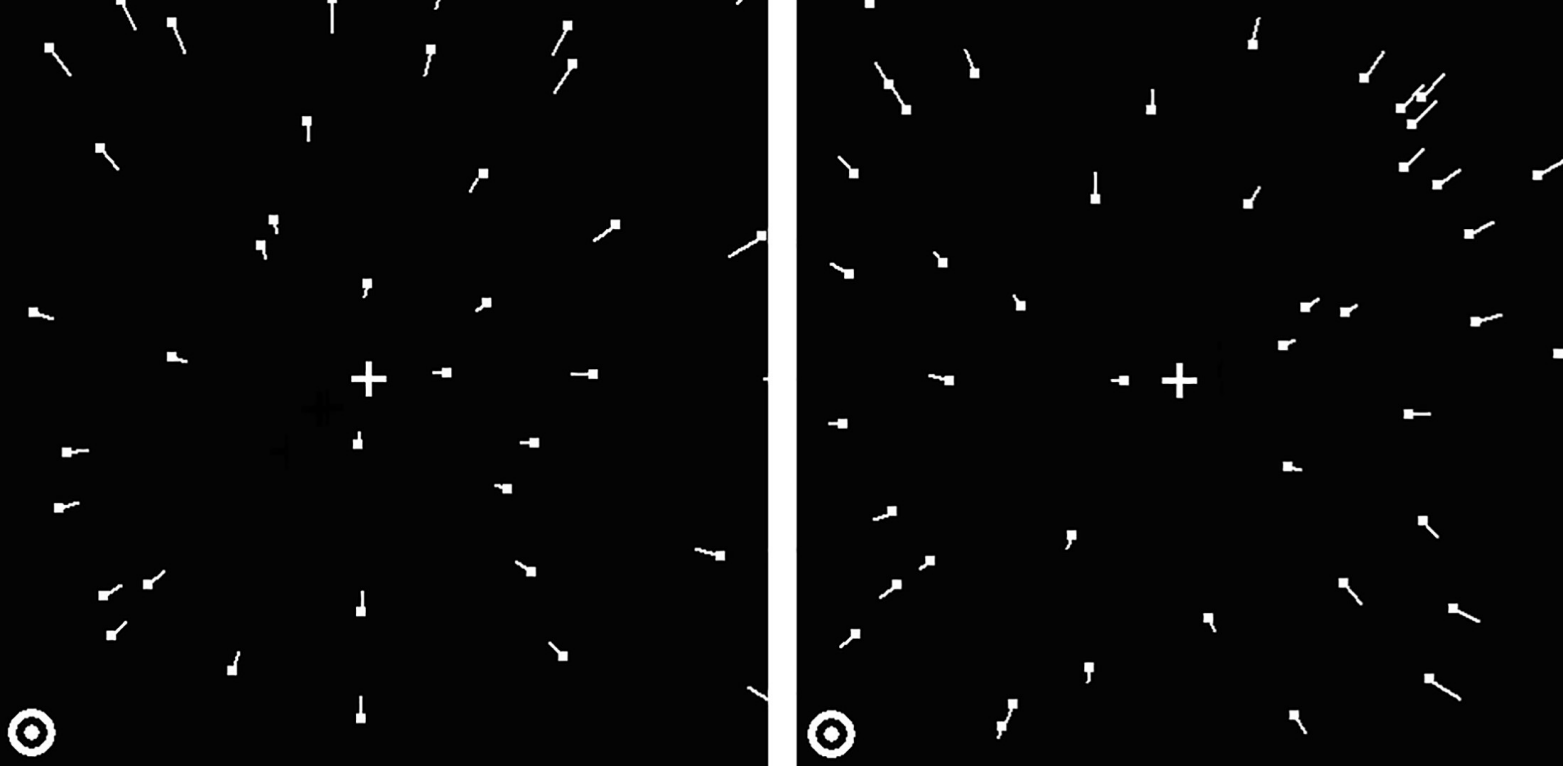
Schematic representations of a contracting (left panel) and an expanding (right panel) optic flow pattern in Experiment 1. In this example, both optic flow patterns were presented in the upper right visual quadrant. In the other three conditions, both flow patterns were presented in one of the other three quadrants. Short white lines (not present in the experimental displays) show the direction of the trajectories of the moving dots (dots are shown at the start of their trajectory). The white cross in each panel (not present in the experimental display) denotes the veridical centres of the flow patterns. The circle in the lower left corner of each panel denotes the fixation point.

The stimuli were presented within a square subtending 19 deg wide × 19 deg high for all the experiments of this study, surrounded by a dark area of screen with a luminance of less than 1 cd/m^2^. The stimuli were random-dot kinematograms of radial flows that mimicked the projected retinal image when an observer is smoothly moving with a constant speed of 4.14 m/s, which is within the speed range of typical steady-state running [33], towards or away from a fronto-parallel square surface, 2 metres away from the observer. The corresponding retinal image produced by forward and backward motion is an expanding (Fig 1, left panel) and a contracting (Fig 1, right panel) optic flow field, respectively. We refer to the veridical centre of each optic flow pattern as the FOE (Focus Of Expansion for the expanding pattern) or the FOC (Focus Of Contraction for the contracting pattern). The FOE/FOCs were always located on imaginary diagonal lines oriented at 45 degrees to the left or right of the vertical meridian. These lines passed through the fixation point, which was always in the display center. In Fig 1, the FOE/FOC of the expanding / contracting optic flow is located in the upper-right visual quadrant. The same principle (mutatis mutandis) applies for the other three visual quadrants (upper left, lower left and lower right). Both the expanding and contracting patterns (9.5 deg wide × 9.5 deg high) were presented within the same visual quadrant. The luminances of the background in the rectangular presentation area and that of the dots were 3.75 cd/m^2^ and 80 cd/m^2^ respectively (measured with a Minolta CS-100 Chromameter photometer). The dots were circular points (diameter 0.14 deg). Dot size and dot shape remained constant throughout the presentation. Each optic flow pattern consisted of 35 frames with no inter-frame interval, resulting in a total duration of 350 ms, at the monitor refresh rate of 100 Hz. The average dot density of each pattern was 1.68 dots/deg^2^. The optic flow display was divided into an imaginary grid of 12 × 12 cells to control dot density. For each frame of the flow pattern, a grid-based wrap-around scheme was used, so that dots moving out of the display aperture or dots producing excessive local density in each cell were recreated within other cells of insufficient dot density, with a speed proportional to its distance from the FOE/FOC. Thus, dot densities across the cells of the grid were kept homogeneous during the stimulus presentation. Each flow sequence was generated off-line and stored in memory to be displayed at the appropriate time. The fixation point fell in one corner of the presentation area (see Fig 1).

#### Design

A two-interval, forced-choice procedure was used to measure any perceived displacement between the centres of a target flow pattern and of a reference flow pattern. On one half of the trials (sub-condition 1), an expanding flow pattern was the target with a contracting one as the reference, and vice versa for the other half (sub-condition 2). Perceived displacements of centre of flow were values averaged across sub-conditions 1 & 2. For each sub-condition, a within-participants design was used with a method of constant stimuli. The distance between the fixation point and the FOE/FOC of the target pattern was always 6.72 deg. The physical offset between the FOE/FOC of the reference pattern and that of the target pattern could take one of 7 possible values (0, ±0.5, ±1.25, and ±2.5 deg). The interval containing the target pattern on any particular trial was randomly chosen. The task was to indicate which interval contained an optic flow pattern with its FOE/FOC closer to the fixation point by pressing one of two response keys. The target and reference patterns were each presented for 350 ms. The last frame of the first pattern was displayed for 100 ms, and then automatically changed into the first frame of the second pattern. This method is similar to the one used in our previous study [16] that aims to avoid the masking effect of the second pattern on the first, while minimizing the decay of the memory of the FOE/FOC of the first pattern. No participant reported problems in remembering the FOE/FOC of the first pattern or any masking effects, when asked at the end of the experiment.

Each physical offset level was presented 20 times, resulting in a total of 1120 trials for Experiment 1 (140 trials for each of two sub-conditions in each of the four visual quadrants). Presentations in the same visual quadrant were randomly mixed, and the total experiment was divided into 16 sub-sessions (four for each visual quadrant with order counterbalanced across participants). Each sub-session contained 70 trials, with a rest period of 1 minute between sub-sessions. The whole experiment took about 60 minutes.

#### Procedure

The observer initiated the experiment by pressing a key. At the beginning of each sub-session, they were told in which quadrant the stimuli would be presented. A red fixation point (radius 0.3 deg) appeared in the centre of the screen for most of the trial duration. Observers were instructed to fixate that point throughout the experiment. For each trial, the fixation point was presented for 300ms before the two moving patterns were displayed sequentially, with the last frame of the second pattern remaining static on the screen. The observer’s response cleared the screen (including the fixation point), and started the next trial after an inter-trial interval of 1 s. Before the formal test, observers were given a set of 20 practice trials with feedback. In the formal experiments, no feedback was provided.

### Results and discussion

#### A significant perceived displacement in the lower-right visual quadrant

**Fig 2:**
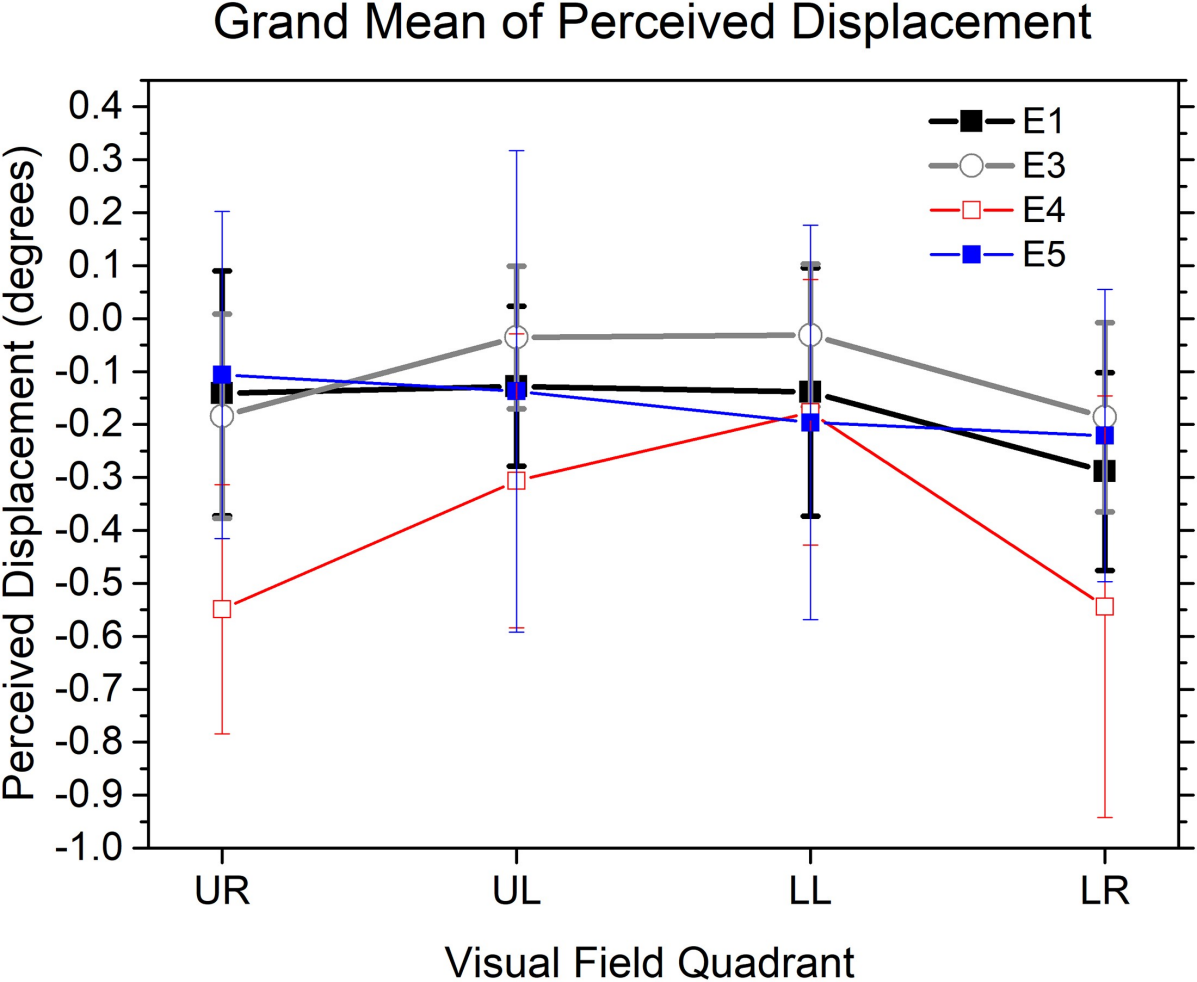
Mean perceived displacements between the centres of two optic flow patterns in each visual quadrant in Experiment 1, 3, 4 & 5. UR: upper right; UL: upper left; LL: lower left; LR: lower right. Experiment 1: E1, black curve. Experiment 3: E3, gray curve. Experiment 4: E4, red curve. Experiment 5: E5, blue curve. Negative values of the y-axis show that the FOE of an expanding flow pattern is perceived as closer to the fixation point than the physically aligned FOC of a contracting flow pattern. Vertical bars represent 95% confidence intervals.

The raw data of each observer were fitted by a logistic function to calculate the 50% points (PSEs). Since the physical position of the target pattern was always fixed, the PSE for the target pattern represents how much shift is needed for the reference pattern to null the perceived displacement between target and reference when they are actually in the same physical position. The PSE was thus a measure of the displacement in perceived centre of flow of the target and reference patterns. Positive values (see Fig 2) indicate that the FOC of the contracting optic flow is perceived closer to the fixation point than the FOE of the expanding optic flow, while negative values mean that the FOE of the expanding optic flow pattern seems closer than the FOC of the contracting flow pattern. The black curve in Fig 2 shows the overall mean perceived flow centre displacement in each visual quadrant. Two analyses were carried out to explore two separate issues- whether these perceived displacements were statistically reliable (i.e., significantly different from zero) in each visual quadrant and how these perceived shifts might be different across the horizontal and vertical meridians of the visual field.

One sample t-tests showed that the perceived displacement of the flow centres was significantly different from zero in the lower right visual quadrant (Mean = -0.29 deg, t(19) = -3.240, p = 0.004 (Bonferroni corrected, the criterion for significance is 0.05/4 = 0.0125), but not in the other three (upper right (t(19) = -1.275, p = 0.218), upper left (t(19) = -1.772, p = 0.092) and lower left (t(19) = -1.238, p = 0.231)) visual quadrants. Thus, in the lower right visual quadrant, the FOE of the expanding optic flow pattern is perceived about 0.3 degrees closer to the fixation point than the FOC of the contracting flow pattern, i.e. an illusory flow centre shift in the direction of the dots in the less eccentric half of the display. There is no significant perceived displacement in the other three visual quadrants.

#### Centre of flow discrimination thresholds

**Fig 3:**
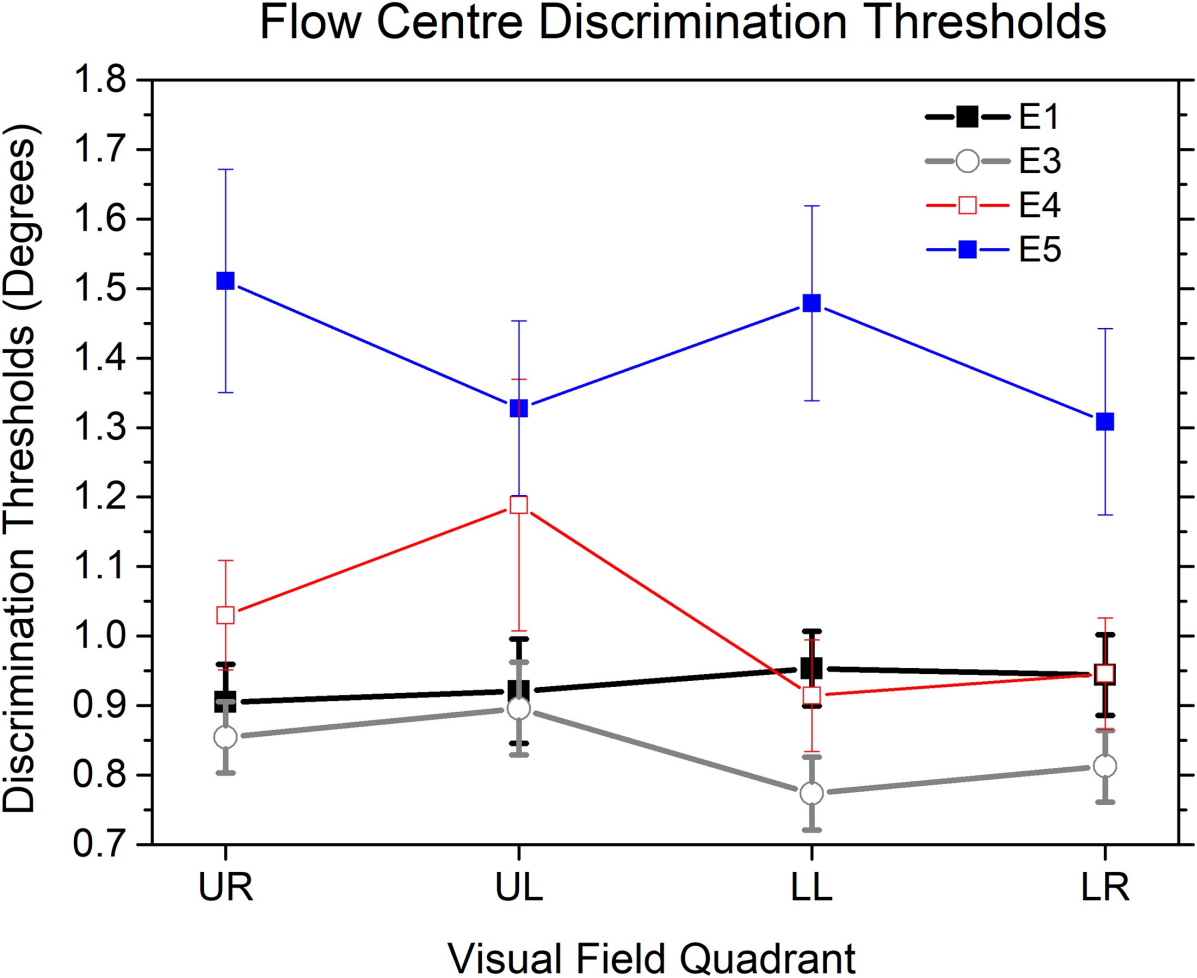
Mean thresholds for discrimination between the centres of two optic flow patterns in each visual quadrant in Experiment 1, 3, 4 & 5. UR: upper right; UL: upper left; LL: lower left; LR: lower right. Experiment 1: E1, black curve. Experiment 3: E3, gray curve. Experiment 4: E4, red curve. Experiment 5: E5, blue curve. Vertical bars represent standard errors.

We calculated the precision of participants’ performance in the flow centre discrimination task in each visual quadrant from their discrimination thresholds (half of the difference in flow centre offset angles between the 25% and 75% points on the fitted psychometric function). The black curve in Fig 3 shows discrimination thresholds for each visual quadrant in Experiment 1. A one-way ANOVA on the thresholds showed no significant main effect of quadrant (F (3, 57) = 0.287, p = .834).

We also calculated the visual hemifield differences in discrimination thresholds. Our data suggested that there was no significant difference of discrimination thresholds between the lower and upper visual hemifields (t (19) = 0.78, p = 0.44) in Experiment 1.

Experiment 1 demonstrated that the FOE of an expanding optic flow pattern is perceived as spatially displaced from the physically superimposed FOC of a contracting flow pattern. This shift was observed only for patterns presented in the lower right visual quadrant, and is not due to differences in the sensitivities of the visual quadrants, since discrimination thresholds are similar everywhere in the visual field.

## Experiment 2

The design of Experiment 1 did not allow us to determine whether there were differences in the precision of discrimination of flow centre in expanding and contracting patterns. This was measured in Experiment 2, which included two conditions. In the first condition, the two patterns were both expanding radial flows (referred to as the EXP-EXP condition), and in the second both were contracting radial flows (the CON-CON condition). An additional aim was to explore possible anisotropies in the precision of discrimination of centre of flow in different regions of the visual field. We expected that there would be no perceived spatial displacements in either condition (unless there was some artifact in Experiment 1 that we had not identified).

### Materials and methods

Seventeen right-handed observers, 12 females and 5 males, aged from 19 to 23, drawn from the same population as Experiment 1, took part in the experiment, and were paid for their participation.

#### Apparatus and stimuli

The apparatus was the same as in Experiment 1. The stimuli of this experiment were exactly the same as in Experiment 1 except that both the target and the reference were either two expanding patterns (the EXP-EXP condition) or two contracting patterns (the CON-CON condition). The target was distinguished from the reference in that the FOE/FOC of the target pattern was kept constant relative to the fixation point, whereas the FOE/FOC of the reference pattern varied relative to that of the target pattern (and to the fixation point). The manipulation on the physical offset between the FOE/FOC of the reference pattern and that of the target pattern was the same as in Experiment 1.

#### Design & procedure

The design was the same as in Experiment 1, except that both optic flow patterns were either expanding patterns or contracting patterns. The procedure was the same as in Experiment 1.

### Results and discussion

#### Perceived displacements of centre of flow

**Fig 4:**
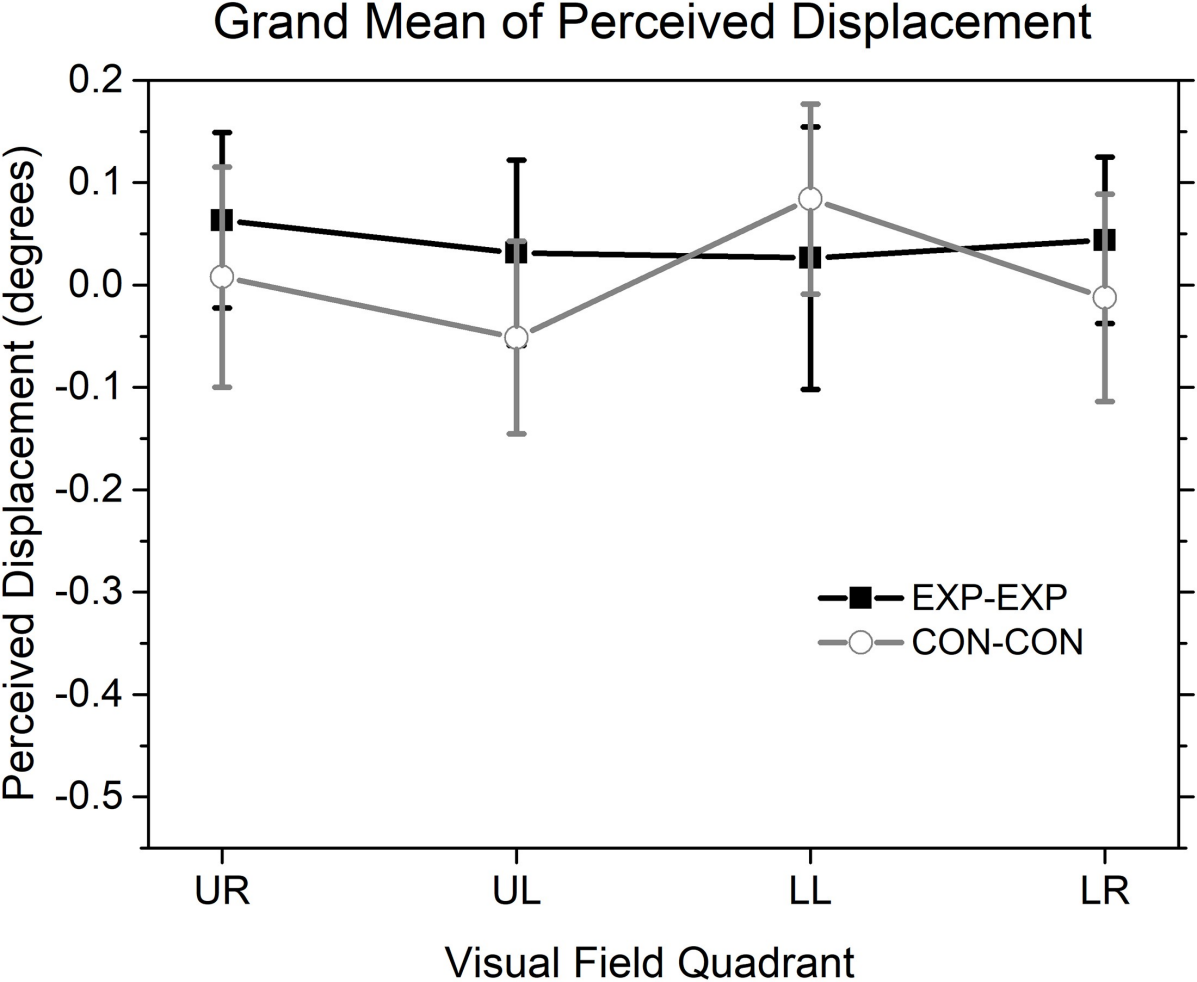
Mean perceived displacements between centres of flow of two patterns in each visual quadrant in the two sub-conditions (EXP-EXP and CON-CON) of Experiment 2. UR: upper right; UL: upper left; LL: lower left; LR: lower right. Vertical bars represent 95% confidence intervals. Negative values of the y-axis show that the FOE or FOC of the target pattern is perceived as closer to the fixation point than the aligned FOE or FOC of the reference pattern. The other details are as in Fig 2.

The perceived displacements between the centres of flow of the target and the reference patterns in each visual quadrant in Experiment 2 are plotted in Fig 4 (the black curve for the EXP-EXP condition and the gray curve for the CON-CON condition). For these two conditions, a positive value of the y-axis indicates that the FOE/FOC of the target flow was perceived as closer to the fixation point than the FOE/FOC of the reference flow. A two-way repeated ANOVA (Flow Direction × Quadrant) showed no significant main effect of either quadrant (F (3, 48) = 0.669, p = 0.575) or flow type (F (1, 16) = 2.106, p = 0.166) and also no significant interaction (F (3, 48) = 0.764, p = 0.520). Further one sample t-tests showed that the perceived flow centre displacements between the target and the reference in Experiment 2 were not significantly different from zero in any given visual quadrant for either the EXP-EXP condition (p = .137, .471, .670 and .271 for the upper right, the upper left, the lower left and the lower right quadrant, respectively; Bonferroni corrected, the criterion for significance is 0.05/4 = 0.0125) or the CON-CON condition (p = .880, .266, .074 and .799 for the upper right, the upper left, the lower left and the lower right quadrant, respectively; Bonferroni corrected, the criterion for significance is 0.05/4 = 0.0125).

#### Discrimination thresholds for centres of flow

**Fig 5:**
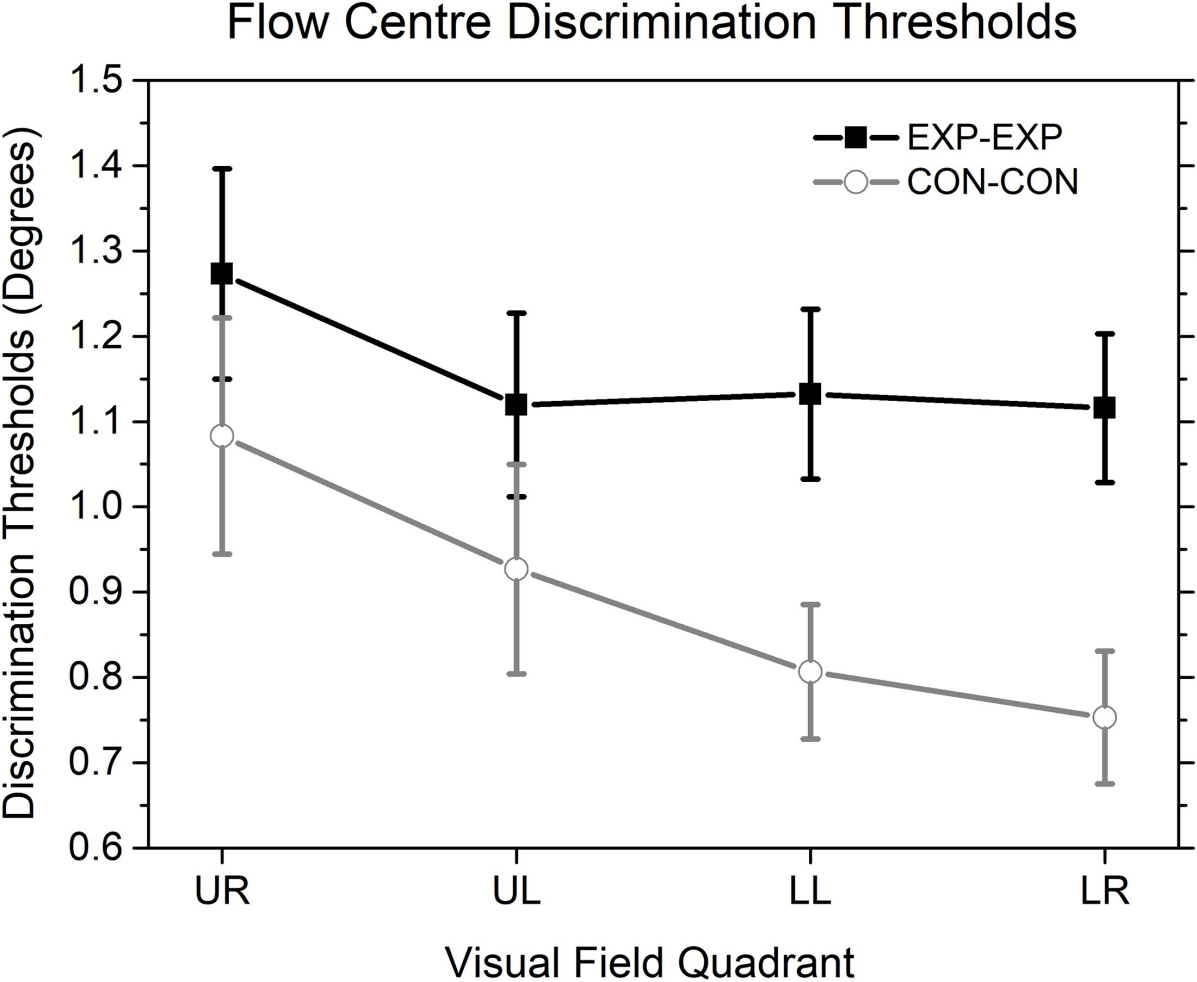
Mean thresholds for discriminating between the positions of the centres of two optic flow patterns in each visual quadrant in the two sub-conditions (EXP-EXP and CON-CON) of Experiment 2. UR: upper right; UL: upper left; LL: lower left; LR: lower right. Vertical error bars represent standard errors. The other details are as in Fig 3.

Discrimination thresholds for centres of flow were also calculated for each visual quadrant in the EXP-EXP condition (the black curve in Fig 5) and the CON-CON condition (the gray curve in Fig 5). A two-way repeated measures ANOVA (Flow Direction × Quadrant) showed significant main effects of both quadrant (F (3, 48) = 4.652, p<0.007) and flow direction (F (1, 16) = 12.733, p<0.004), but no significant interactions (F (3, 48) = 0.633, p = 0.597). Bonferroni corrected pairwise comparisons showed that discrimination thresholds were smaller, after averaging the data across all visual quadrants, for the CON-CON condition (p <.004; difference = -0.268 deg, SE = 0.075 deg) than for the EXP-EXP condition. Thresholds averaged across the EXP-EXP and the CON-CON conditions were significantly smaller in the lower right than in the upper right visual quadrant (p <.018; difference = -0.244 deg, SE = 0.069 deg) and there was no significant difference between any other pairs of quadrants. Further Bonferroni corrected pairwise comparisons suggested that discrimination thresholds in the EXP-EXP condition were significantly higher than those in the CON-CON condition in the lower visual hemifield, including the lower left (p <.006; difference = 0.325 deg, SE = 0.101 deg) and the lower right (p <.001; difference = 0.363 deg, SE = 0.069 deg) visual quadrants, but not in the upper visual hemifield, including the upper left (p = 0.133) and the upper right (p = 0.294) visual quadrants.

Experiment 2 showed that there are differences in the precision of discrimination of the centres of optic flow patterns that depend both on the type of pattern and position in the visual field. Precision is higher for contracting than expanding patterns, an effect that is larger in the lower visual hemifield. Experiment 2 also showed that discrimination thresholds were smaller in the lower right than in the upper right visual quadrant. As expected, there were no significant shifts in perceived centres of flow in any visual quadrant when the two flow patterns were identical.

Putting the results of Experiments 1 & 2 together, it appears that the shift of perceived centre of flow in the lower right visual quadrant observed in Experiment 1 is a genuine effect rather than an artifact induced by confounding variables, such as a difference in the precision of discrimination for different visual quadrants or for different types of optic flow. There are two main reasons for this conclusion. First, one can see that the pattern of perceived displacements observed in Experiment 1 is qualitatively different from that of the precision of discriminations observed in Experiment 2. Second, if the shift observed in Experiment 1 were due to a higher sensitivity to the centre of the contracting than of the expanding flow pattern in the lower right visual quadrant, we would expect to find the same flow centre shift in the lower left visual quadrant, since Experiment 2 demonstrated that the sensitivity to the differences in flow centres between two flow types are similar in the lower left and the lower right visual quadrants. However, the non-significant shift in the lower left visual quadrant demonstrated in Experiment 1 is not consistent with this account.

## Experiment 3

In Experiment 1, the display mimicked the pattern of stimulation produced by an observer’s motion towards and away from a single fronto-parallel surface. However, as suggested by Cutting and Wang [34], in the real world the observer might make judgments about the centre of flow based on depth information from multiple objects located at different distances. For example, Ito & Shibata [35] showed that when expanding and contracting dot fields are superimposed, but presented at different distances, it is the further one that determines the direction of perceived self-motion. However, because it is not clear how the introduction of multiple depth planes might affect performance on our tasks, we ran a further experiment. In Experiment 3, the same type of dot fields were used as in Experiment 1, but the velocities of some of the dots were altered, to mimic those that would be produced by movement towards or away from multiple fronto-parallel surfaces. The aim was to determine whether spreading information about centres of flow across different depth planes would improve the precision of discrimination and alter the pattern of perceived displacements.

### Materials and methods

Twenty-one right-handed observers, 16 females and 5 males, aged from 19 to 25, drawn from the same population as before, took part in the experiment, and were paid for their participation.

#### Apparatus and stimuli

**Fig 6:**
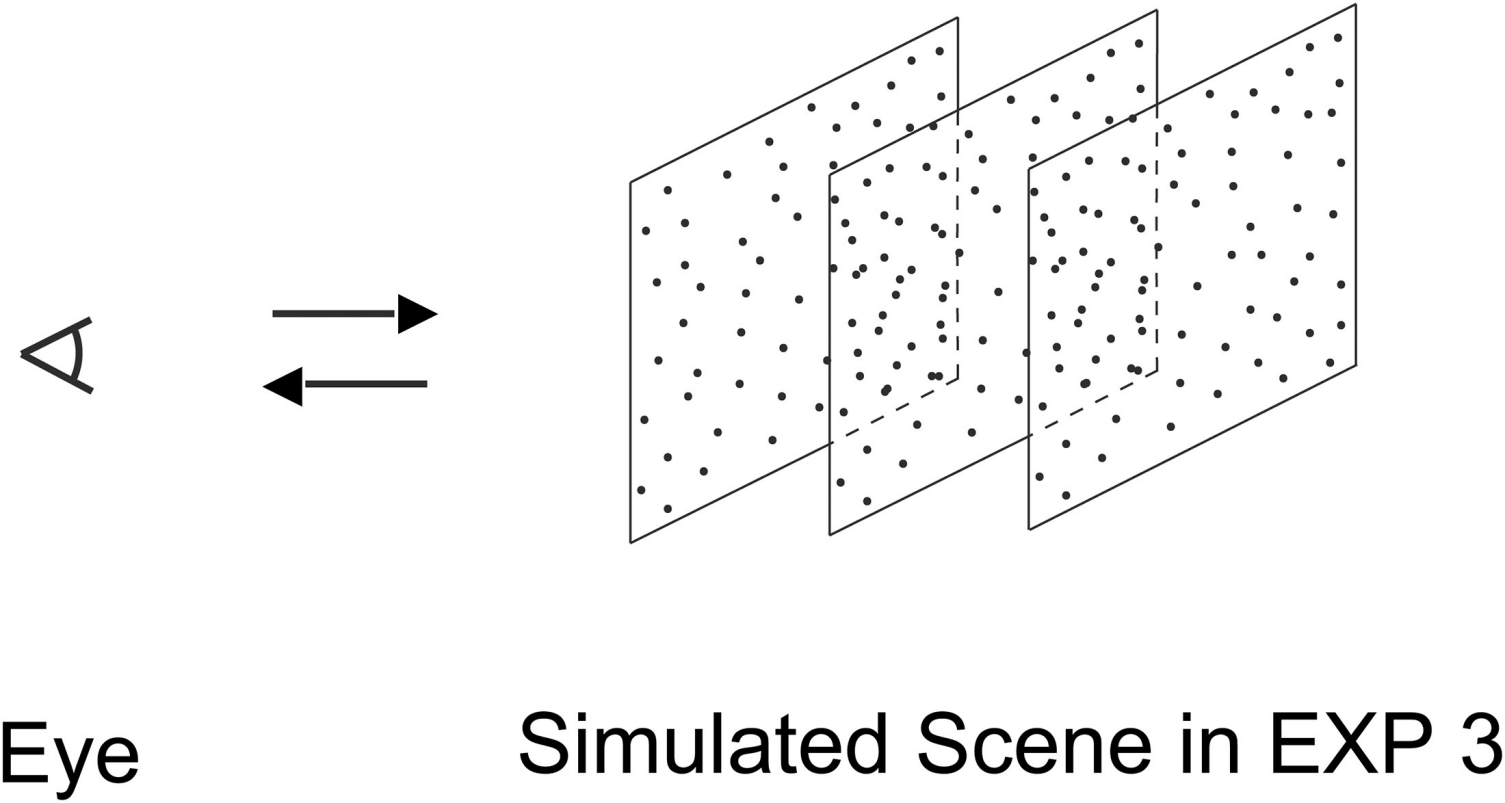
Schematic representation of the simulated scene in Experiment 3, which mimicked the effects of an observer’s motion towards or away from three transparent frontoparallel surfaces covered with random dots. Experiments 1, 2, 4 & 5 mimicked an observer’s smooth motion towards or away from a single frontoparallel surface. The single frontoparallel surface was located at a distance from the observer’s eye equal to the middle surface in this figure for Experiments 1, 2 & 5 and equal to the left surface in this figure for Experiment 4, respectively. The average dot density on the single frontoparallel surface was three times of and equal to that on a surface in this figure for Experiments 1, 2 & 4 and Experiment 5, respectively.

The apparatus was the same as in Experiment 1. Depth information was introduced by a method similar to that used by Royden & Hildreth [36, 37] in a heading discrimination task. The stimuli of Experiment 3 were the same as in Experiment 1 except that the trajectories of individual dots were those that would be produced if they lay on one of three front-parallel square surfaces, which were 1.25m, 2.0m and 2.75m away from the observers, respectively (see Fig 6 for a graphical representation of the simulated scene). The average dot density on each surface was one third of that on the single surface in Experiment 1. The same grid-based wrap-around scheme as in Experiment 1 was used for each surface, resulting in a dot density matched to that in the displays in Experiment 1 & 2. The observers reported that the dot fields appeared three-dimensional.

#### Design & procedure

The design and procedure were the same as in Experiment 1.

### Results and discussion

#### Perceived displacements of flow centres

The perceived displacements of centres of flow in each visual quadrant in Experiment 3 are plotted as the gray curve in Fig 2. One sample t-tests suggested that perceived displacement was not significantly different from zero in any of the four visual quadrants, including the lower right (t(20) = -2.172, p = 0.042), upper right (t(20) = -1.988, p = 0.061), upper left (t(20) = -0.399, p = 0.694 and lower left (t(20) = -0.488, p = 0.631) visual quadrants (Bonferroni corrected, the criterion for significance is 0.05/4 = 0.0125). Thus, there was no significant perceived displacement between the FOE and the FOC in any of the four visual quadrants. In particular, the perceived displacement observed in Experiment 1 was reduced to a non-significant level in Experiment 3 by the multiple speed gradients.

#### Discrimination thresholds

The gray curve in Fig 3 shows the discrimination threshold for each visual quadrant in Experiment 3. A one-way ANOVA on the thresholds showed no significant main effect of quadrant (F (3, 60) = 2.162, p = .102). Comparisons between data from Experiment 1 and data from Experiment 3 suggested that the discrimination threshold of Experiment 3 was lower relative to that of Experiment 1 only in the lower left visual quadrant (t (39) = -2.40, p<0.022, difference = -0.180 deg), but not in the other three visual quadrants (upper right (p = 0.500), upper left (p = 0.805) and lower right (p = 0.099)).

As in Experiment 1, we also calculated the visual hemifield differences in discrimination thresholds. Our data showed that discrimination threshold was significantly larger (difference = 0.08 deg) in the upper than that in the lower visual hemifield (t (20) = 2.25, p<0.05) in Experiment 3. This effect was a critical departure from that of Experiment 1.

The results of Experiment 3 suggested that the introduction of extra depth information altered the pattern of displacement of perceived centres of flow. Unlike in Experiment 1, the FOE of an expanding flow pattern in Experiment 3 was not perceived as displaced from the FOC of a contracting optic flow pattern in the lower right visual quadrant. This suggests that human participants might be able to use the extra global information from the multiple speed gradients to minimize the perceived bias in the lower visual hemifield. This idea was supported by changes in the discrimination thresholds, which were significantly reduced in the lower visual hemifield relative to those in the upper visual hemifield when multiple speed gradients were used in Experiment 3, but not in Experiment 1 when a single speed gradient was used.

## Experiment 4

In Experiment 3, the perceived bias for localization of the expansion centres relative to the contraction centres was absent when we used stimuli with multiple speed gradients to simulate locomotion towards or away from three fronto-parallel surfaces located at different distances from the observers. We suggested that the perceived bias in the lower right visual quadrant in Experiment 1 was likely speed gradient-sensitive and could be reduced to a non-significant level by the usage of multiple speed gradients in Experiment 3. However, an alternative explanation to these results could be that the difference in results between Experiments 1 & 3 was possibly not due to the presence of multiple gradients in Experiment 3 per se, but rather, to the fact that one of the gradients from the simulated surface closest to the observers had overall faster speeds than the speeds generated for the single plane in Experiment 1. Would it be possible that the trajectories of the moving dots with higher overall speeds than that in Experiment 1 increased the observers’ precision of spatial localization and reduced the perceived bias into a non-significant level? Here, we carried out a control experiment by the usage of stimuli with higher overall trajectory speeds to explore this issue.

### Materials and methods

Twenty right-handed observers, 19 females and 1 male, aged from 18 to 24, drawn from the same population as before, took part in the experiment, and were paid for their participation.

#### Apparatus and stimuli

The apparatus was the same as in Experiment 1. The stimuli of this experiment were the same as in Experiment 1 except that the random dots of radial flows lay on a single front-parallel surface 1.25m away from the observers (a distance same as the left surface in Fig 6), i.e., 0.75m closer to the observers than that in Experiment 1 (the middle surface in Fig 6), and thus produced the trajectories of the moving dots with higher overall speeds than that in Experiment 1. The average dot density on the single surface was equal to that in Experiment 1.

#### Design & procedure

The design and procedure were the same as in Experiment 1.

### Results and discussion

#### Perceived displacements of flow centres

The perceived displacements of centres of flow in each visual quadrant in Experiment 4 are plotted as the red curve in Fig 2. One sample t-tests suggested that perceived displacements were significantly different from zero in the upper right (Mean = -0.55 deg, t(19) = -4.878, p<0.001) and lower right (Mean = -0.54 deg, t(19) = -2.862, p = 0.010; Bonferroni corrected, the criterion for significance is 0.05/4 = 0.0125) visual quadrants, but not in the upper left (t(19) = -2.311, p = 0.032) and lower left (t(19) = -1.478, p = 0.156) visual quadrants. Thus, in both the lower right and upper right visual quadrants, the FOE of the expanding optic flow pattern is perceived about 0.5 degrees closer to the fixation point than the FOC of the contracting flow pattern. There is no significant perceived displacement in the other two visual quadrants.

#### Discrimination thresholds

The red curve in Fig 3 shows the discrimination threshold for each visual quadrant in Experiment 4. A one-way ANOVA on the thresholds showed no significant main effect of quadrant (F (1.686, 32.027) = 2.678, p = .092; After Greenhouse-Geisser correction). Comparisons between data from Experiment 4 and data from Experiment 1 suggested that the discrimination thresholds of Experiment 4 were not significantly different from those of Experiment 1 in each of the four visual quadrants (ps>.05).

As in Experiment 1, we also calculated the visual hemifield differences in discrimination thresholds. Our data showed that discrimination thresholds were not significantly different between the upper and the lower visual hemifields (t (19) = 1.845, p = 0.081) in Experiment 4, an effect similar to that in Experiment 1.

The results of Experiment 4 did not support the hypothesis that the trajectories of the moving dots with higher overall speeds might reduce the perceived bias for localization of the centres of the flow patterns (see Fig 2) or increase the observers’ precision of spatial localization (see Fig 3). However, we did observe that the perceived spatial bias of localization was modulated by the overall speeds of the trajectories of the moving dots. Particularly, compared the results in Experiment 4 with that in Experiment 1, the perceived bias in the upper right visual quadrant in Experiment 4 was contingent upon overall speeds of the trajectories of the moving dots, i.e., reduced to a non-significant level in Experiment 1 by the usage of relatively lower overall speeds of the trajectories (see Fig 2). In contrast, the perceived displacement in the lower right visual quadrant was relatively robust and present consistently for both high and low overall speeds of the trajectories in Experiment 4 and Experiment 1 (see Fig 2).

Combining the results of Experiment 1, 3 & 4 together, we suggested that the perceived displacements in Experiment 4 may have different origins for the upper right and lower right visual quadrants. The bias in the lower right visual quadrant observed in both Experiment 1 & 4 was likely speed gradient-sensitive and could be reduced to a non-significant level by the usage of multiple speed gradients. On the contrary, the perceived bias in the upper right visual quadrant observed in Experiment 4 was possibly overall speed-sensitive and modulated by the overall speeds of the trajectories of the moving dots. The removal of the perceived shift of centres of flow in the upper right visual quadrant from Experiment 4 to Experiment 3 was likely due to a reduction of the overall speeds of the trajectories between these two experiments. Here, the overall speeds of the trajectories of the moving dots was calculated based on an average of all three simulated surfaces in Experiment 3, which should be smaller than the overall speeds of the trajectories calculated based on the simulated single surface in Experiment 4. In contrast, the removal of the perceived shift of centres of flow in the lower right visual quadrant from Experiment 4 to Experiment 3 was likely due to the usage of multiple speed gradients, rather than a reduction of the overall speeds of the trajectories between these two experiments. Above explanations are consistent with the fact that the reduction of the overall speeds of the trajectories in Experiment 4 per se, relative to Experiment 1, removed the perceived bias in the upper right visual quadrant but not in the lower right visual quadrant.

## Experiment 5

A key difference between Experiment 1 and Experiment 3 was that the average dot density on the single simulated surface in Experiment 1 was three times greater than the average dot density on each of the three simulated surfaces in Experiments 3. Thus, another alternative explanation on the difference between the results of these two experiments could be that the observers’ spatial localization in Experiment 3 was based on predominant processing on one of the three simulated surfaces with an average dot density one third as that in Experiment 1. The combination of the predominant single surface and its lower average dot density might result in the elimination of perceived bias on the estimation of centres of expansion/contraction in Experiment 3 relative to that in Experiment 1. Here, we carried out another control experiment by simulating a single surface with lower average dot density to test this ‘single surface with lower average dot density hypothesis’.

### Materials and methods

Twenty right-handed observers, 18 females and 2 males, aged from 18 to 27, drawn from the same population as before, took part in the experiment, and were paid for their participation.

#### Apparatus and stimuli

The apparatus was the same as in Experiment 1. The stimuli of this experiment were the same as in Experiment 1 except that the average dot density on the single surface was one third of that in Experiment 1.

#### Design & procedure

The design and procedure were the same as in Experiment 1.

### Results and discussion

#### Perceived displacements of flow centres

The perceived displacements of centres of flow in each visual quadrant in Experiment 5 are plotted as the blue curve in Fig 2. One sample t-tests suggested that perceived displacements were not significantly different from zero in the upper right (t (19) = -0.720, p = 0.48), upper left (t (19) = -0.633, p = 0.534), lower left (t (19) = -1.101, p = 0.284) and lower right (t (19) = -1.676, p = 0.110) visual quadrants. Thus, there is no significant perceived displacement in any of the four visual quadrants.

#### Discrimination thresholds

The blue curve in Fig 3 shows the discrimination threshold for each visual quadrant in Experiment 5. A one-way ANOVA on the thresholds showed no significant main effect of quadrant (F (2.060, 39.132) = 1.680, p = .199; After Greenhouse-Geisser correction). Comparisons between the data from Experiment 5 and that from Experiment 3 suggested that the discrimination thresholds of Experiment 5 were significantly larger than that of Experiment 3 in each of the four visual quadrants, including the upper right (t (22.86) = 3.892, p = 0.001, difference = 0.656 deg, equal variances not assumed), upper left (t (39) = 3.076, p = 0.004, difference = 0.432 deg), lower left (t (24.222) = 4.720, p = 0.001, difference = 0.705 deg, equal variances not assumed) and lower right (t (24.472) = 3.448, p = 0.002, difference = 0.496 deg, equal variances not assumed) visual quadrants.

As in Experiment 1, we also calculated the visual hemifield differences in discrimination thresholds. Our data showed that discrimination thresholds were not significantly different between the upper and the lower visual hemifields (t (19) = 0.219, p = 0.829) in Experiment 5, an effect similar to that in Experiment 1.

The only difference between Experiment 5 and Experiment 1 was that the average dot density of Experiment 5 was reduced into one third of that in Experiment 1. The perceived bias in the lower right visual quadrant observed in Experiment 1 (the black curve in Fig 2) was reduced to non-significant level in Experiment 5 (the blue curve in Fig 2). Thus, the results of these two experiments did provide partial support to the ‘single surface with lower average dot density hypothesis’ in that the reduction of the average dot density would reduce or even remove the perceived bias on the estimation of centres of expansion/contraction with decreased flow information. However, the results did not support the other part of the ‘single surface with lower average dot density hypothesis’ which suggested that the observers’ spatial localization in Experiment 3 was based on predominant processing on one single simulated surface rather than on all three simulated surfaces as a whole. If the other two simulated surfaces had no substantial contributions to the observers’ performance in Experiment 3, we would expect that the valid dot density in Experiment 3, which was calculated based on a surface processed predominantly by the observers over the other two surfaces, matched with the valid dot density in Experiment 5, which was calculated based on the only surface in the visual field. The matched valid dot densities would render the precisions of the spatial localization comparable between Experiment 3 & 5. However, we found that the localization precision of Experiment 5 (the blue curve in Fig 3) was significantly worse than that of Experiment 3 (the gray curve in Fig 3) in each of the four visual quadrants. Taken together, these results suggested that it was unlikely that the observers’ spatial localizations were based on processing to one speed gradient singled out from all three ones. Instead, it was more likely that a holistic processing was adopted to process the three simulated surfaces as a whole and to extract information from all available speed gradients in the visual field simultaneously. This may support an approach to calculate the valid average dot density based on summation of all simulated surfaces.

## General discussion

Our previous studies [15, 16] demonstrated that in the lower right visual quadrant a contour defined by a diverging pattern is perceived as more eccentric than one defined by a converging pattern. We suggested that this displacement in the lower right visual quadrant is related to the greater sensitivity to centripetal motion in human peripheral vision [17–19]. According to this explanation, the nature of the spatial displacements of motion-defined contours is that a region in centripetal motion (e.g. the less eccentric part of a diverging pattern) is perceptually expanded into that containing centrifugal motion (e.g. the more eccentric region of a diverging pattern).

However, in Experiment 4 of the present study, when an expanding and a contracting optic flow pattern were presented sequentially in the lower right and upper right visual quadrant, the perceived shifts of their centres of flow were contrary to predictions based on the ‘centripetal bias’, in that the centre of an expanding pattern was perceived as closer to, not further away from, the fovea than that of a contracting pattern. Further experiments suggested that the perceived displacements in Experiment 4 may have different origins for the upper right and lower right visual quadrants. The bias in the lower right visual quadrant observed in both Experiment 1 & 4 was likely speed gradient-sensitive, i.e. could be reduced to a non-significant level by the multiple speed gradients in Experiment 3. On the contrary, the perceived bias in the upper right visual quadrant observed in Experiment 4 was possibly overall speed-sensitive and removed by a reduction of the overall speeds of the trajectories of the moving dots in Experiment 1. Below, we discuss why the difference between the two types of stimulus patterns and the difference between the two biases in the lower right and upper right visual quadrants might have occurred and the potential reason for the effect of the speed gradient(s) in the lower right visual quadrant and the effect of the overall speeds of the trajectories in the upper right visual quadrant.

### Locations defined by opposing translations and by radial flow

One potentially important difference between the two types of stimuli (diverging/converging translation, and radial expansion/contraction) is the region of the total pattern needed to locate the junction and the centre of flow, respectively. Imagine that most of a radial pattern was obscured, leaving visible only a small region nearest to the fovea. Although the centre of flow would no longer be visible, it could be located by extrapolating back along the trajectories and changes of velocity (speed gradients) of dots in the visible region of the pattern. However, for contours defined by translations in opposite directions, this would not be possible: at least part of the region on both sides of the contour would be necessary to locate it.

This analysis appears consistent with the results of Experiments 1 & 4. In these experiments, the directions of the shifts of perceived centres of flow (a centripetal shift for the FOE and a centrifugal shift for the FOC) were in the same direction as the motion signals in the less eccentric region of the pattern, e.g. the part of the flow field between the fovea and the FOE/FOC. Thus, it seems that the direction of the perceived displacement was governed by the less eccentric region of the flow patterns. In contrast, locating a contour defined by opposing translations requires the pattern regions on both sides of the contour, especially those near to the contour, to be processed simultaneously. It is on such a local process that we suppose the ‘centripetal bias’ to act.

Experiment 3 provides support to our explanation of the role of speed gradients in the centre of a flow discrimination task, since it introduced multiple speed gradients. This led to two differences from Experiment 1 in which a single speed gradient was used. The first effect was a more precise discrimination of the centres of flow in the lower, relative to the upper, visual hemifield. The other effect was a removal of the perceived shift of centres of flow in the lower right visual quadrant. Previous neurophysiological work [38] demonstrated that neurons in Area MST, which are supposed to process optic flow, have clear preferences for stimuli with a speed gradient (slower speeds in the centre, faster in the periphery) over those with no speed gradient. Putting Duffy & Wurtz’s finding and our Experiment 3 together, it is very likely that neurons in Area MST are not only selective to speed-gradient but also highly sensitive to number of speed-gradients, and benefit from the presence of multiple speed-gradient cues.

### Upper vs. lower and left vs. right visual fields

The upper visual hemifield has an advantage in tasks requiring the discrimination of fine detail and local processing, such as local feature detection [39], word recognition [40], size judgments of stationary lines [41], and face-sex categorization [42, 43]. Because of the pattern of mislocalisations of contours defined by opposing translations (greatest in the lower visual hemifield), we suggested that their localization might be a local process [15, 16].

However, in the current study, the sensitivity of discrimination of centre of flow is higher in the lower than in the upper visual hemifield (see the E3 condition in Fig 3 and the CON-CON condition in Fig 5), as would be expected if most information about centre of flow in natural scenes comes from the ground plane. This is consistent with the hypothesis that the lower visual hemifield has an advantage in processing global features [39] since previous researchers have demonstrated that humans rely on the global radial structure of the flow pattern to extract translational heading [28–30, 37, 44–53], rather than on the local centre of expansion / contraction. This idea is supported by evidence that the receptive fields processing optic flow are much larger than those handling simple translation [22, 24, 25], and that perception of the centre of flow in heading tasks is possible when it is hidden [45] or when dots with random directions are introduced into the radial pattern [54, 55]. Thus, our findings are consistent with a hypothesis that the localization of motion-defined contours is largely a local process, while discrimination of centres of flow is reliant on more global information integration across parts of the visual field. In the lower visual hemifield, the localization of flow centres is more susceptible to unbalanced flow pattern sampling. This is due to the fact that the near side of a flow pattern is more close to the fovea and thus sampled more than those of the far side of the flow, resulting in a shifted FOE/FOC after integration of samples, with its direction consistent with motion signals of the near side flow pattern. This bias was consistent with previous finding [39] indicating that the lower, relative to the upper, visual hemifield plays a special role during global processing. Additional global information, such as that provided by multiple speed gradients in the lower visual hemifield is able to improve the discrimination of centres of flow, resulting in increased sensitivity and a non-significant level of perceived bias. Above account explains why we did not observe a significant perceived flow centre shift in the lower right visual quadrant in Experiment 3 as that in Experiment 1, i.e., a speed gradient-sensitive bias in the lower right visual quadrant. The results of Experiment 5 also provided support to the global processing hypothesis in that a holistic processing on multiple speed gradients, rather than a predominant processing on a single speed gradient, was adopted during the localization of a flow centre.

Experiment 4 demonstrated another type of perceived bias in the upper right visual quadrant which showed a property, i.e., sensitivity to the overall speeds of the trajectories of the moving dots, that was absent for the bias in the lower right visual quadrant. We suspect that this new type of bias reflects another origin of the unbalanced flow pattern sampling during the localization of the flow centre, i.e., a hemispheric asymmetry in attentional allocation in favor of motion-induced spatial displacement in the right visual hemifield, which is different from the bias induced by global processing more strongly linked with the lower visual hemifield. As suggested by Heilman & Van Den Abell [20] and exemplified by the phenomenon of pseudoneglect, the left visual hemifield has a superiority during the allocation of attentional resource, which makes it less susceptible to spatial illusions induced by either local or global processing. This idea is consistent with the absence of perceived bias in the left visual hemifield in the current study with the usage of the optic flow patterns and the absence of perceived bias in the lower right visual quadrant in our earlier study [16] with the usage of the motion-defined contours.

On the contrary, the less attentional resource allocated to the right visual hemifield makes it more susceptible to motion-induced spatial illusions, such as the one induced by a global processing to localize the centre of a flow. Particularly, it is highly possible that the lack of attentional resource may introduce new unbalanced flow sampling in the right hemifield by having the near side of a flow pattern sampled more than the far side of the flow. This is perhaps due to the fact that the near side of the flow is closer to the fovea and thus more efficient to capture attention than the far side of the flow. It is not surprising that the unbalanced flow sampling induced by the hemispheric asymmetry in attentional allocation is likely modulated by the overall speeds of the trajectories of the moving dots since the dots with higher overall speeds are more efficient to capture attention in general. Above accounts on the second origin of the perceived bias are consistent with our findings in Experiment 1 & 4. Namely, the perceived bias in the upper right visual quadrant was overall speed-sensitive, i.e., present for a condition with higher overall speeds in Experiment 4 and absent for a condition with lower overall speeds in Experiment 1. However, because the stimuli and paradigm used here are different from those in the typical study of pseudoneglect [20, 56], the hypothesis of the asymmetry in attentional allocation requires confirmation, perhaps by explicitly manipulating spatial attention.

### Expanding vs. contracting radial flow

The direction of shift in perceived centre of flow in the lower right and upper right visual quadrants in Experiment 1 & 4 is consistent with previous studies [29, 52] demonstrating a perceived shift of heading towards the fixation point during forward locomotion with peripheral viewing. However, these studies did not compare displays that would be produced by backwards as well as forwards locomotion. Our findings suggest that the difference between expanding and contracting patterns entails not only a perceived shift of centre of flow, as demonstrated in our Experiments 1 & 4, but also in the precision of flow centre discrimination, as suggested by Kerzel and Hecht [57] and our Experiment 2. The greater precision in discrimination of flow centres for the contracting pattern that we found is also consistent with previous work [58], which demonstrated lower motion coherence thresholds for radially contracting than for expanding patterns. The present study added new support to the idea that many factors contribute to the accuracy of determining the focus of an expanding/contracting optic flow pattern. Those factors include the eccentricity of the centre of the stimulus relative to the fovea, i.e. the retinal eccentricity [45, 52, 59], the eccentricity of the focus of the flow pattern relative to the centre of the stimulus, i.e. the heading eccentricity [45], the stimulus presentation duration [60], the motion direction-related flow type, i.e. expansion vs. contraction [57], as well as the effect of different visual hemifields demonstrated by this study.

### Vision in natural scenes

Given the nature of our task (to judge the relative positions of two flow centres rather than a standard heading discrimination task), and the characteristics of the stimuli used, (briefly (350 ms) and relatively small (9.5 x 9.5 deg) optic flow patterns), it is unlikely that self-motion, instead of object motion, played a dominant role in the effect we found. In particular, our results suggested that this effect can be influenced by speed gradients. However, it is still possible that shifts in apparent centre of flow occur and can influence perception of heading, at least when viewing impoverished displays in which multiple speed gradients are either unavailable or distorted by eye movements. It is uncertain how far other factors, such as changes of fixation and shifts of attention, and cues such as the relative motion between objects in real scenes, can interact with shifts in perceived centre of flow in tasks such as driving, in which heading accuracy of about 1 degree is needed to avoid collisions [61]. The shifts we measured were smaller than this, but occurred with full attention, which is unlikely to be maintained during a lengthy drive in a vehicle. Experiments in a driving simulator would be required to address these questions.

## Conclusion

In conclusion, we found shift in perceived centre of flow in peripherally viewed expanding and contracting optic flow patterns, which was either speed gradient-sensitive or overall speed-sensitive and varied for different regions of the visual field. Our data suggest that this variation might reflect a combination of two effects: an advantage of global processing in favor of the lower visual hemifield and a hemispheric asymmetry in attentional allocation in favor of motion-induced spatial displacement in the right visual hemifield. These anisotropies may introduce errors in localizing the centres of 2-D optic flow patterns in peripheral vision.
